# Worse characteristics can predict survival effectively in bilateral primary breast cancer: A competing risk nomogram using the SEER database

**DOI:** 10.1002/cam4.2662

**Published:** 2019-10-30

**Authors:** Kaiwen Shen, Longdi Yao, Jinli Wei, Zhou Luo, Wang Yu, Huamin Zhai, Jianwen Wang, Luhong Chen, Deyuan Fu

**Affiliations:** ^1^ Yangzhou University Medical Academy Yangzhou Jiangsu China; ^2^ The Second Clinical College of Dalian Medical University Dalian Liaoning China; ^3^ Department of Thyroid and Breast Surgery Yangzhou University Affiliated Northern Jiangsu People's Hospital Yangzhou Jiangsu China; ^4^ Department of Thyroid and Breast Surgery The Second Xiangya Hospital of Central South University Changsha Hunan China

**Keywords:** bilateral breast neoplasm, end results (SEER) database, epidemiology, nomograms, prognosis, surveillance

## Abstract

**Objective:**

There is limited information from population‐based cancer registries regarding prognostic features of bilateral primary breast cancer (BPBC).

**Methods:**

Female patients diagnosed with BPBC between 2004 and 2014 were randomly divided into training (n = 7740) and validation (n = 2579) cohorts from the Surveillance, Epidemiology, and End Results Database. We proposed five various models. Multivariate Cox hazard regression and competing risk analysis were to explore prognosis factors in training cohort. Competing risk nomograms were constructed to combine significant prognostic factors to predict the 3‐year and the 5‐year survival of patients with BPBC. At last, in the validation cohort, the new score performance was evaluated with respect to the area under curve, concordance index, net reclassification index and calibration curve.

**Results:**

We found out that age, interval time, lymph nodes invasion, tumor size, tumor grade and estrogen receptor status were independent prognostic factors in both multivariate Cox hazard regression analysis and competing risk analysis. Concordance index in the model of the worse characteristics was 0.816 (95% CI: 0.791‐0.840), of the bilateral tumors was 0.819 (95% CI: 0.793‐0.844), of the worse tumor was 0.807 (0.782‐0.832), of the first tumor was 0.744 (0.728‐0.763) and of the second tumor was 0.778 (0.762‐0.794). Net reclassification index of the 3‐year and the 5‐year between them was 2.7% and −1.0%. The calibration curves showed high concordance between the nomogram prediction and actual observation.

**Conclusion:**

The prognosis of BPBC depended on bilateral tumors. The competing risk nomogram of the model of the worse characteristics may help clinicians predict survival simply and effectively. Metachronous bilateral breast cancer presented poorer survival than synchronous bilateral breast cancer.

## Graphical abstract

This study aimed to find out prognostic factors and put forward a new prognostic model in bilateral primary breast cancer from the Surveillance, Epidemiology, and End Results Database. We demonstrated that the new prediction was as effective as the model of bilateral tumors, however, more simply and convenient.

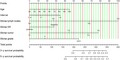

## 1 INTRODUCTION

Breast cancer is the most common female malignancy worldwide^1^ and the contralateral primary breast cancer is the most common second primary cancer in breast cancer patients.^2^, ^3^ Nowadays, the increasing breast cancer incidence rates, improving diagnosis and longer life expectancy have contributed to the growing number of female patients at risk for bilateral primary breast cancer (BPBC), which comprised of approximately 2%‐11% of all breast cancer.^4^, ^5^ Most studies reported that patients diagnosed with contralateral breast cancer had worse prognosis than patients with unilateral breast cancer (UBC).^4^, ^6^, ^7^, ^8^, ^9^ However, little is known that what significant factors lead to worse prognosis in patients with BPBC and their impact on prognosis is controversial. We have no idea whether it is the first tumor, the second tumor or the bilateral tumors which plays a more important role in BPBC.

With the rapid development of early detection and treatment, the mortality has decreased greatly in developed countries.^10^, ^11^ Nevertheless, a corollary of reduced mortality is the greater opportunity to come to being other conditions, such as second primary cancer and cardiovascular disease.^12^ A high risk of competing noncancer events is inevitable. Therefore, it is necessary to consider the competing death when evaluating the prognosis. Nowadays, the competing risk analysis has been widely used in various cancer research.^13^, ^14^, ^15^, ^16^, ^17^, ^18^

Nomogram is a valuable and convenient tool to quantify various biological and clinical variables to generate a graph of mathematical model that can predict a special endpoint.^19^ To date, several competing risk nomograms have been constructed to predict the survival probability for cancers such as thyroid cancer and lung cancer.^17^, ^18^

The purpose of the study was to find out prognostic factors in BPBC by competing risk analysis. Based on this, we looked for a concise model and constructed a competing risk nomogram that could be used for individualized risk assessment in BPBC.

## 2 PATIENTS AND METHOD

### 2.1 Data acquisition and patient selection

The data were selected from 18 registries of the Surveillance, Epidemiology, and End Results Database (SEER) program, which included female patients with breast cancer from 2004 to 2014. Then, we excluded the patients as follows:

1. Follow‐up less than 3 months.
2. Did not undergo a surgical operation.
3. Cancer metastasis.
4. Ductal carcinoma in situ and lobular carcinoma in situ.
5. Unknown data.
6. Unconfirmed pathology.

For obtaining BPBC patients, we then merged patient‐unique identification numbers, removed patients diagnosed with third or more primaries cancer and ipsilateral breast cancer. At last, there were 10 319 BPBC patients included in the study.

The following data were collected for each patient: patient number, age, race, interval time, follow‐up time, death, cancer‐specific death, other causes of death, tumor size, lymph nodes, grade, estrogen receptor (ER) status, progesterone receptor (PR) status, histologic type, radiation record, surgical method. As chemotherapy record was no/unknown and human epidermal growth factor receptor‐2 (HER‐2) status was available after 2010, the data were not incorporated into research and analysis. In addition, age and follow‐up time of contralateral breast cancer were calculated.

### 2.2 Construction of the nomograms

The eligible patients were divided into two groups randomly: training cohort (n = 7740) and validation cohort (n = 2579). Continuous variable (interval time) was classified into three groups with X‐tile. Interval time means the interval between the first primary breast cancer and the second primary breast cancer. We conducted a descriptive analysis of the baseline clinical features of the included patients and used the chi‐square test to compare the characteristics of synchronous bilateral breast cancer (SBBC) and metachronous bilateral breast cancer (MBBC). The multivariable Cox regression analysis was used to define the factors independently influencing breast cancer‐specific survival (BCSS) in the training cohort. Afterwards, some meaningless variables were excluded by stepwise model selection. Based on this, we further screened for prognosis impact factors by Fine and Gray's competing risk regression analysis and constructed a corresponding competing risk nomogram.^20^, ^21^ In addition, there were four new models brought up:

1. The worse characteristics regardless of side (eg if left tumor was 20 mm, 5 positive lymph nodes and ER negative, and right tumor was 50 mm, no positive lymph nodes and ER positive. Fifty millimeter would be selected for size, 5 positive lymph nodes for lymph nodes and ER negative for ER status), including tumor size, lymph nodes, tumor grade, ER status and PR status;
2. The characteristics of worse tumor, based on lymph nodes, then tumor size, then tumor grade, then ER status, and then PR status; This order depended on the hazard by the following analysis.
3. The characteristics of first tumor;
4. The characteristics of second tumor.

### 2.3 Validation of the nomograms

To evaluate the discrimination and accuracy ability of five competing risk nomograms, we used the Harrell's concordance index (C‐index) with a 95% confidence interval (95% CI) in the validation cohort, which were subjected to 500 bootstrap resamples. The value of C‐index ranges from 0.5 to 1, which resembles the area under the curve (AUC).^22^ 0.5 indicates a random chance and 1 reflects a perfect discrimination. Calibration plots (500 bootstrap resamples) were generated to examine the agreement between the nomogram‐predicted and actual 3‐year and 5‐year survival. The predictions were expected to fall on a 45° diagonal line in a perfect calibrated model. Moreover, we drew receiver operating characteristic curves of five models, and made a comparison among them. Net reclassification improvement (NRI, continuous version) was estimated to classify cases and controls adequately for analyzing the predictive abilities between the worse characteristics and the characteristics of bilateral tumors.^23^ NRI = *P* (cases classified better in nomograms) − *P* (cases classified worse in nomograms) + *P* (controls classified better in nomograms) − *P* (controls classified worse in nomograms).

### 2.4 Statistical analyses

Data analyses were performed using R software version 3.5.1 (R Foundation for Statistical Computing) and X‐tile version 3.6.1(Robert L Camp, Yale University). Two‐sided *P* values less than .05 were considered statistically significant.

## 3 RESULTS

### 3.1 Clinicopathologic characteristics of patients

AS shown in Table 1, a total of 10 319 eligible patients from 2004 to 2014 were identified from the SEER database. A quarter of the patients was classified as the validation group randomly, and the rest were used to develop nomograms. The median follow‐up time was 65 months and was calculated for the entire study cohort according to the reverse Kaplan‐Meier method. The median age and interval of all patients was 63 years and 20 months. During the study period, 9.59% patients died from breast cancer and 7.95% patients died from other causes. Patients died of other causes accounted for approximately 45% of all deaths. There were not apparently significant statistical differences between patients in the training and validation cohort except age, tumor size of the first primary breast cancer and histologic of the second primary breast cancer.

**Table 1:** Demographic and clinical characteristics of the included bilateral primary breast cancer patients in the SEER database

Demographic and clinical characteristics	All patients	Training cohort	Validation cohort	*P*‐value
	n = 10 319	n = 7740	n = 2579	
Age (y)	62.98 ± 13.01	63.15 ± 12.95	62.45 ± 13.17	.034
Interval (mo)	20.10 ± 32.55	20.05 ± 32.53	20.27 ± 32.62	.725
Race				.733
White	8491 (82.29%)	6379 (82.42%)	2112 (81.89%)	
Black	972 (9.42%)	719 (9.29%)	253 (9.81%)	
Other	856 (8.30%)	642 (8.29%)	214 (8.30%)	
Marital				.67
Yes	5539 (53.68%)	4164 (53.80%)	1375 (53.32%)	
No	4780 (46.32%)	3576 (46.20%)	1204 (46.68%)	
Interval (mo)				.796
<1	3973 (38.50%)	2989 (38.62%)	984 (38.15%)	
1‐4	2479 (24.02%)	1847 (23.86%)	632 (24.51%)	
>4	3867 (37.47%)	2904 (37.52%)	963 (37.34%)	
First Tumor				
Histologic				.631
IDC	7344 (71.17%)	5494 (70.98%)	1850 (71.73%)	
ILC	2500 (24.23%)	1882 (24.32%)	618 (23.96%)	
Other	475 (4.60%)	364 (4.70%)	111 (4.30%)	
Grade				.144
I	2666 (25.84%)	2007 (25.93%)	659 (25.55%)	
II	4690 (45.45%)	3549 (45.85%)	1141 (44.24%)	
III/IV	2963 (28.71%)	2184 (28.22%)	779 (30.21%)	
Surgery				.56
BCS	4372 (42.37%)	3292 (42.53%)	1080 (41.88%)	
Mastectomy	5947 (57.63%)	4448 (57.47%)	1499 (58.12%)	
Radiation				.763
Yes	4683 (45.38%)	3506 (45.30%)	1177 (45.64%)	
No	5636 (54.62%)	4234 (54.70%)	1402 (54.36%)	
ER				.129
Positive	8606 (83.40%)	6480 (83.72%)	2126 (82.44%)	
Negative	1713 (16.60%)	1260 (16.28%)	453 (17.56%)	
PR				.133
Positive	7725 (74.86%)	5823 (75.23%)	1902 (73.75%)	
Negative	2594 (25.14%)	1917 (24.77%)	677 (26.25%)	
Tumor size				.567
T1	6081 (58.93%)	4568 (59.02%)	1513 (58.67%)	
T2	3083 (29.88%)	2322 (30.00%)	761 (29.51%)	
T3	539 (5.22%)	391 (5.05%)	148 (5.74%)	
T4	616 (5.97%)	459 (5.93%)	157 (6.09%)	
Lymph nodes				.01
N0	6919 (67.05%)	5232 (67.60%)	1687 (65.41%)	
N1	2273 (22.03%)	1647 (21.28%)	626 (24.27%)	
N2	723 (7.01%)	559 (7.22%)	164 (6.36%)	
N3	404 (3.92%)	302 (3.90%)	102 (3.96%)	
Second tumor				
Histologic				.015
IDC	7226 (70.03%)	5397 (69.73%)	1829 (70.92%)	
ILC	2551 (24.72%)	1908 (24.65%)	643 (24.93%)	
Other	542 (5.25%)	435 (5.62%)	107 (4.15%)	
Grade				.534
I	3363 (32.59%)	2545 (32.88%)	818 (31.72%)	
II	4565 (44.24%)	3405 (43.99%)	1160 (44.98%)	
III/IV	2391 (23.17%)	1790 (23.13%)	601 (23.30%)	
Surgery				.038
BCS	3781 (36.64%)	2880 (37.21%)	901 (34.94%)	
Mastectomy	6538 (63.36%)	4860 (62.79%)	1678 (65.06%)	
Radiation				.221
Yes	3386 (32.81%)	2565 (33.14%)	821 (31.83%)	
No	6933 (67.19%)	5175 (66.86%)	1758 (68.17%)	
ER				.256
Positive	8821 (85.48%)	6634 (85.71%)	2187 (84.80%)	
Negative	1498 (14.52%)	1106 (14.29%)	392 (15.20%)	
PR				.876
Positive	7502 (72.70%)	5624 (72.66%)	1878 (72.82%)	
Negative	2817 (27.30%)	2116 (27.34%)	701 (27.18%)	
Tumor size				.859
T1	7852 (76.09%)	5900 (76.23%)	1952 (75.69%)	
T2	2036 (19.73%)	1524 (19.69%)	512 (19.85%)	
T3	297 (2.88%)	218 (2.82%)	79 (3.06%)	
T4	134 (1.30%)	98 (1.27%)	36 (1.40%)	
Lymph nodes				.428
N0	8188 (79.35%)	6163 (79.63%)	2025 (78.52%)	
N1	1530 (14.83%)	1128 (14.57%)	402 (15.59%)	
N2	370 (3.59%)	271 (3.50%)	99 (3.84%)	
N3	231 (2.24%)	178 (2.30%)	53 (2.06%)	
The cause of death				.917
Survival	8509 (82.46%)	6378 (82.40%)	2131 (82.63%)	
Breast	990 (9.59%)	742 (9.59%)	248 (9.62%)	
Other	820 (7.95%)	620 (8.01%)	200 (7.75%)	

Note

In “the causes of death”, “survival” means that patients are alive. “Breast” means that patients die attributing to breast cancer. “Other” means that patients die attributing to other causes except breast cancer.

Abbreviations: BCS, breast‐conserving surgery; ER, estrogen receptor; IDC, infiltrating ductal carcinoma; ILC, infiltrating lobular carcinoma; PR, progesterone receptor; SEER, Surveillance, Epidemiology and End Results.

In Table 2, included patients were divided into two groups based on synchronous (interval ≤ 4 months) and metachronous (interval > 4 months) bilateral breast cancer, and there were distinct differences between two groups (*P* < .001). Patients with MBBC tended to be older, more often infiltrating ductal carcinoma, the worse differentiated grade and had a higher proportion of ER and PR negative status both in the first tumor (11.11% vs 25.76%, 19.51% vs 34.52%, *P* < .001) and the second tumor (8.40% vs 24.72%, 18.03% vs 42.77%, *P* < .001). Besides, in contrast to patients with SBBC, ER, PR discordance and ER, PR concordant negativity made up a larger proportion in MBBC (*P* < .001).

**Table 2:** Demographic and clinical characteristics between SBBC and MBBC

Demographic and clinical characteristics	All patients	Interval ≤4	Interval >4	*P*‐value
	N = 10 319	N = 6452	N = 3867	
Age		61.78 ± 12.75	64.99 ± 13.20	<.001
ER				<.001
+/+	7843 (76.01%)	5504 (85.31%)	2339 (60.49%)	
+/−	735 (7.12%)	311 (4.82%)	424 (10.96%)	
−/+	763 (7.39%)	231 (3.58%)	532 (13.76%)	
−/−	978 (9.48%)	406 (6.29%)	572 (14.79%)	
PR				<.001
+/+	7075 (68.56%)	4993 (77.39%)	2082 (53.84%)	
+/−	848 (8.22%)	342 (5.30%)	506 (13.09%)	
−/+	650 (6.30%)	200 (3.10%)	450 (11.64%)	
−/−	1746 (16.92%)	917 (14.21%)	829 (21.44%)	
First tumor				
Histologic				<.001
IDC	7344 (71.17%)	4376 (67.82%)	2968 (76.75%)	
ILC	2500 (24.23%)	1807 (28.01%)	693 (17.92%)	
Other	475 (4.60%)	269 (4.17%)	206 (5.33%)	
Grade				<.001
I	2666 (25.84%)	1740 (26.97%)	926 (23.95%)	
II	4690 (45.45%)	3110 (48.20%)	1580 (40.86%)	
III/IV	2963 (28.71%)	1602 (24.83%)	1361 (35.20%)	
Surgery				<.001
BCS	4372 (42.37%)	1894 (29.36%)	2478 (64.08%)	
Mastectomy	5947 (57.63%)	4558 (70.64%)	1389 (35.92%)	
Radiation				<.001
Yes	4683 (45.38%)	2349 (36.41%)	2334 (60.36%)	
No	5636 (54.62%)	4103 (63.59%)	1533 (39.64%)	
ER				<.001
Positive	8606 (83.40%)	5735 (88.89%)	2871 (74.24%)	
Negative	1713 (16.60%)	717 (11.11%)	996 (25.76%)	
PR				<.001
Positive	7725 (74.86%)	5193 (80.49%)	2532 (65.48%)	
Negative	2594 (25.14%)	1259 (19.51%)	1335 (34.52%)	
ER/PR				<.001
+/+	7638 (74.02%)	5156 (79.91%)	2482 (64.18%)	
+/− or −/+	1055 (10.22%)	616 (9.55%)	439 (11.35%)	
−/−	1626 (15.76%)	680 (10.54%)	946 (24.46%)	
Tumor				<.001
T1	6081 (58.93%)	3577 (55.44%)	2504 (64.75%)	
T2	3083 (29.88%)	2068 (32.05%)	1015 (26.25%)	
T3	539 (5.22%)	381 (5.91%)	158 (4.09%)	
T4	616 (5.97%)	426 (6.60%)	190 (4.91%)	
Lymph nodes				<.001
N0	6919 (67.05%)	4082 (63.27%)	2837 (73.36%)	
N1	2273 (22.03%)	1580 (24.49%)	693 (17.92%)	
N2	723 (7.01%)	512 (7.94%)	211 (5.46%)	
N3	404 (3.92%)	278 (4.31%)	126 (3.26%)	
Second tumor				
Histologic				<.001
IDC	7226 (70.03%)	4329 (67.10%)	2897 (74.92%)	
ILC	2551 (24.72%)	1790 (27.74%)	761 (19.68%)	
Other	542 (5.25%)	333 (5.16%)	209 (5.40%)	
Grade				<.001
I	3363 (32.59%)	2405 (37.28%)	958 (24.77%)	
II	4565 (44.24%)	2976 (46.13%)	1589 (41.09%)	
III/IV	2391 (23.17%)	1071 (16.60%)	1320 (34.13%)	
Surgery				<.001
BCS	3781 (36.64%)	1979 (30.67%)	1802 (46.60%)	
Mastectomy	6538 (63.36%)	4473 (69.33%)	2065 (53.40%)	
Radiation				<.001
Yes	3386 (32.81%)	1985 (30.77%)	1401 (36.23%)	
No	6933 (67.19%)	4467 (69.23%)	2466 (63.77%)	
ER				<.001
Positive	8821 (85.48%)	5910 (91.60%)	2911 (75.28%)	
Negative	1498 (14.52%)	542 (8.40%)	956 (24.72%)	
PR				<.001
Positive	7502 (72.70%)	5289 (81.97%)	2213 (57.23%)	
Negative	2817 (27.30%)	1163 (18.03%)	1654 (42.77%)	
ER/PR				<.001
+/+	7420 (71.91%)	5256 (81.46%)	2164 (55.96%)	
+/− or −/+	1483 (14.37%)	687 (10.65%)	796 (20.58%)	
−/−	1416 (13.72%)	509 (7.89%)	907 (23.45%)	
Tumor				.002
T1	7852 (76.09%)	4977 (77.14%)	2875 (74.35%)	
T2	2036 (19.73%)	1225 (18.99%)	811 (20.97%)	
T3	297 (2.88%)	181 (2.81%)	116 (3.00%)	
T4	134 (1.30%)	69 (1.07%)	65 (1.68%)	
Lymph nodes				<.001
N0	8188 (79.35%)	5170 (80.13%)	3018 (78.04%)	
N1	1530 (14.83%)	954 (14.79%)	576 (14.90%)	
N2	370 (3.59%)	215 (3.33%)	155 (4.01%)	
N3	231 (2.24%)	113 (1.75%)	118 (3.05%)	

Abbreviations: BCS, breast‐conserving surgery; ER, estrogen receptor; IDC, infiltrating ducal carcinoma; ILC, infiltrating lobular carcinoma; MBBC, metachronous bilateral breast cancer; PR, progesterone receptor; SBBC, synchronous bilateral breast cancer.

### 3.2 Screening for prognostic factors

The multivariate Cox analyses of the bilateral tumors in BCSS were listed in Table 3. After stepwise model selection, we excluded bilateral histologic, PR status and surgery of the first tumor. Race and radiation of the first tumor were not found to be independently predictive of survival (*P* = .221 and *P* = .057, respectively). The strongest predictors were age at diagnosis, interval, ER status, tumor size, lymph nodes, and tumor grade (*P* < .001). Increasing age per year was predictive of worsened survival (hazards ratio [HR]:1.015; 95% CI: 1.009‐1.021; *P* < .0001). In order to rule out the influence of competing death events, we carried out multivariable competing risk analysis to identify the following independent prognostic factors: age, interval, ER status, tumor grade, tumor size and lymph nodes (*P* < .05).

**Table 3:** Multivariate COX and competing risk analysis of bilateral tumors in BPBC

Variable	Multivariate analysis		Multivariable competing risk analysis	
	HR (95% CI)	*P*‐value	SHR (95% CI)	*P*‐value
Age	1.0149 (1.009‐1.021)	<.001	1.009 (1.003‐1.02)	.004
Race				
White	Reference			
Black	1.1422 (0.923‐1.413)	.221	1.067 (0.838‐1.36)	.600
Other	0.7319 (0.534‐1.003)	.053	0.742 (0.537‐1.02)	.070
Marital				
Yes	Reference			
No	1.212 (1.042‐1.409)	.013	1.136 (0.972‐1.33)	.110
Interval				
<1	Reference			
1‐4	0.9024 (0.726‐1.123)	.356	0.898 (0.719‐1.12)	.340
>4	1.6416 (1.380‐1.954)	<.001	1.530 (1.269‐1.84)	<.001
First tumor				
Grade				
I	Reference			
II	1.125 (0.890‐1.422)	.324	1.107 (0.877‐1.4)	.390
III/IV	1.4952 (1.163‐1.923)	.002	1.433 (1.108‐1.85)	.006
Radiation				
Yes	Reference			
No	1.1805 (0.995‐1.401)	.057	1.150 (0.964‐1.37)	.120
ER				
Positive	Reference			
Negative	1.3568 (1.147‐1.605)	<.001	1.36 (1.139‐1.62)	.001
Tumor				
T1	Reference			
T2	1.5941 (1.328‐1.914)	<.001	1.575 (1.313‐1.89)	<.001
T3	1.6852 (1.257‐2.260)	.001	1.655 (1.199‐2.28)	.002
T4	2.7461 (2.137‐3.529)	<.001	2.421 (1.845‐3.18)	<.001
Lymph nodes				
N0	Reference			
N1	1.5343 (1.267‐1.859)	<.001	1.503 (1.238‐1.82)	<.001
N2	2.4671 (1.957‐3.111)	<.001	2.465 (1.926‐3.16)	<.001
N3	3.6732 (2.841‐4.749)	<.001	3.735 (2.805‐4.97)	<.001
Second tumor				
Grade				
I	Reference			
II	1.1744 (0.955‐1.445)	.128	1.157 (0.941‐1.42)	.170
III/IV	1.6937 (1.343‐2.136)	<.001	1.683 (1.322‐2.14)	<.001
Surgery				
BCS	Reference			
Mastectomy	0.8206 (0.679‐0.991)	.040	0.865 (0.708‐1.06)	.150
Radiation				
Yes	Reference			
No	1.2394 (1.019‐1.508)	.032	1.158 (0.940‐1.43)	.170
ER				
Positive	Reference			
Negative	1.3874 (1.170‐1.645)	<.001	1.366 (1.138‐1.64)	.001
Tumor				
T1	Reference			
T2	1.4378 (1.208‐1.712)	<.001	1.373 (1.146‐1.64)	.001
T3	1.5503 (1.121‐2.144)	.008	1.468 (1.02‐2.11)	.039
T4	1.5146 (1.020‐2.250)	.040	1.518 (0.993‐2.32)	.054
Lymph nodes				
N0	Reference			
N1	1.5064 (1.242‐1.827)	<.001	1.511 (1.238‐1.84)	<.001
N2	2.2529 (1.714‐2.962)	<.001	2.144 (1.567‐2.93)	<.001
N3	3.4334 (2.559‐4.606)	<.001	3.168 (2.285‐4.39)	<.001

Note

After stepwise model selection, we excluded bilateral histologic, PR and surgery of first tumor.

Abbreviations: BCS, breast‐conserving surgery; BPBC, bilateral primary breast cancer; ER, estrogen receptor; PR, progesterone receptor.

We performed the same statistical analysis above in four new models. In Table 4, Stepwise model selection eliminated surgery and histologic. Under multivariable Cox regression analysis, radiation of the second tumor and the worse PR status were not to be independently predictive of survival. After multivariable competing risk analysis, not surprisingly, we sought out the same independent prognostic factors: age, interval, ER status, tumor grade, tumor size and lymph nodes (*P* < .05). Interval of 1‐4 months showed better survival than interval less than 1 month in multivariable cox regression (HR: 0.819; *P* = .032) and competing risk analysis (HR: 0.818; *P* = .069). Multivariable Cox regression and competing risk analysis of the rest models were shown in Tables [Link], [Link], [Link].

**Table 4:** Multivariate COX and competing risk analysis of worse characteristics regardless of side in BPBC

Variable	Multivariate analysis		Multivariable competing risk analysis	
	HR (95% CI)	*P*‐value	SHR (95% CI)	*P*‐value
Age	1.013 (1.008‐1.018)	<.001	1.007 (1.001‐1.01)	.018
Race				
White	Reference		Reference	
Black	1.301(1.087‐1.558)	.0041	1.151 (0.869‐0.917)	.230
Other	0.766(0.583‐1.006)	.0551	0.757 (0.552‐1.04)	.085
Marital				
Yes	Reference		Reference	
No	1.268 (1.112‐1.445)	<.001	1.139 (0.979‐1.32)	.092
Interval				
<1	Reference		Reference	
1‐4	0.819 (0.682‐0.983)	.032	0.818 (0.658‐1.02)	.069
>4	1.375 (1.182‐1.600)	<.001	1.434 (1.192‐1.73)	<.001
First radiation				
Yes	Reference		Reference	
No	1.208 (1.048‐1.392)	.009	1.168 (0.992‐1.38)	.062
Second radiation				
Yes	Reference		Reference	
No	1.103 (0.950‐1.282)	.1989	1.076 (0.905‐1.28)	.110
Worse tumor				
T1	Reference		Reference	
T2	2.130 (1.782‐2.546)	<.001	2.002 (1.626‐2.46)	<.001
T3	2.971 (2.351‐3.756)	<.001	2.821 (2.142‐3.71)	<.001
T4	3.866 (3.098‐4.823)	<.001	3.302 (2.509‐4.35)	<.001
Worse lymph nodes				
N0	Reference		Reference	
N1	1.523 (1.283‐1.808)	<.001	1.548 (1.268‐1.89)	<.001
N2	3.127 (2.581‐3.788)	<.001	3.049 (2.419‐3.84)	<.001
N3	5.597 (4.585‐6.832)	<.001	5.238 (4.110‐6.68)	<.001
Grade				
I	Reference		Reference	
II	1.415 (1.030‐1.945)	.0324	1.325 (0.935‐1.88)	.110
III/IV	2.457 (1.783‐3.385)	<.001	2.342 (1.643‐3.34)	<.001
Worse ER				
Positive	Reference		Reference	
Negative	1.477 (1.237‐1.763)	<.001	1.457 (1.190‐1.78)	<.001
Worse PR				
Positive	Reference		Reference	
Negative	1.157 (0.964‐1.389)	.1183	1.126 (0.916‐1.38)	.260

Note

After stepwise model selection, we excluded bilateral surgery and histologic.

Abbreviations: BCS, breast‐conserving surgery; BPBC, bilateral primary breast cancer; ER, estrogen receptor; PR, progesterone receptor.

### 3.3 Developing competing risk nomograms

**Figure 1:**
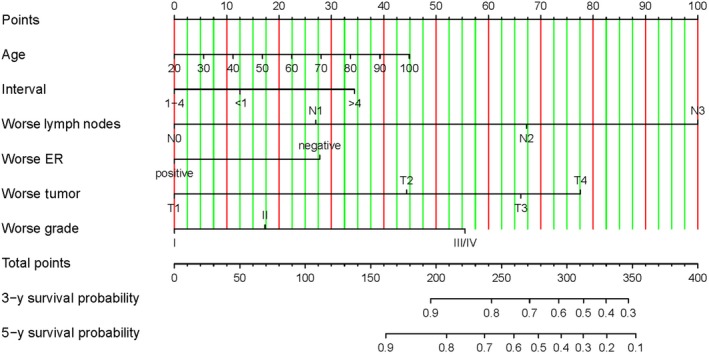
Nomogram for predicting the 3‐ and 5‐y survival probabilities of worse characteristics regardless of side in bilateral primary breast cancer. A vertical straight line was drawn from the variable value to the axis labeled “Points” to identify points for each variable. All points were summed, and the total points was projected to the scales along the bottom of the figure that correspond to the 3‐ and 5‐y survival. Notes: ER, estrogen receptor

Considering the outcomes of the included variables in five models, the competing risk nomograms were constructed to predict the 3‐ and 5‐year survival (Figure 1; Figures [Link], [Link], [Link], [Link]). By adding up the scores corresponding to each value and normalizing the total scores to the baseline scale, we can easily estimate the predictors for the 3‐ and 5‐year survival.

### 3.4 Calibration and validation of the nomograms

**Figure 2:**
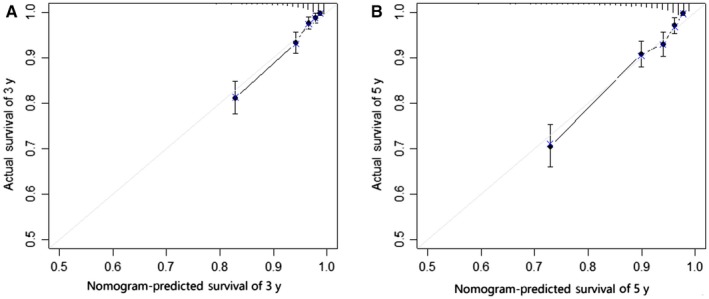
Calibration curves for predicting the 3‐y (A) and 5‐y (B) survival of bilateral tumors in the validation cohort

**Figure 3:**
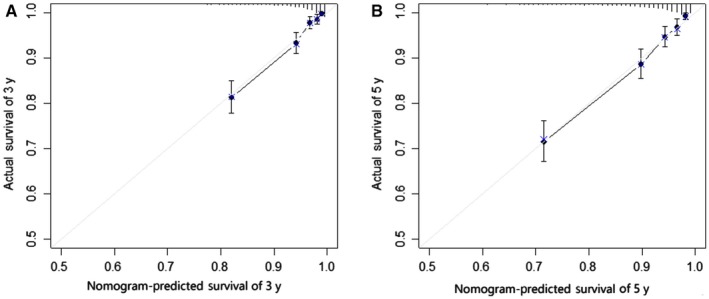
Calibration curves for predicting the 3‐y (A) and 5‐y (B) survival of worse characteristics regardless of side in bilateral primary breast cancer in the validation cohort

**Figure 4:**
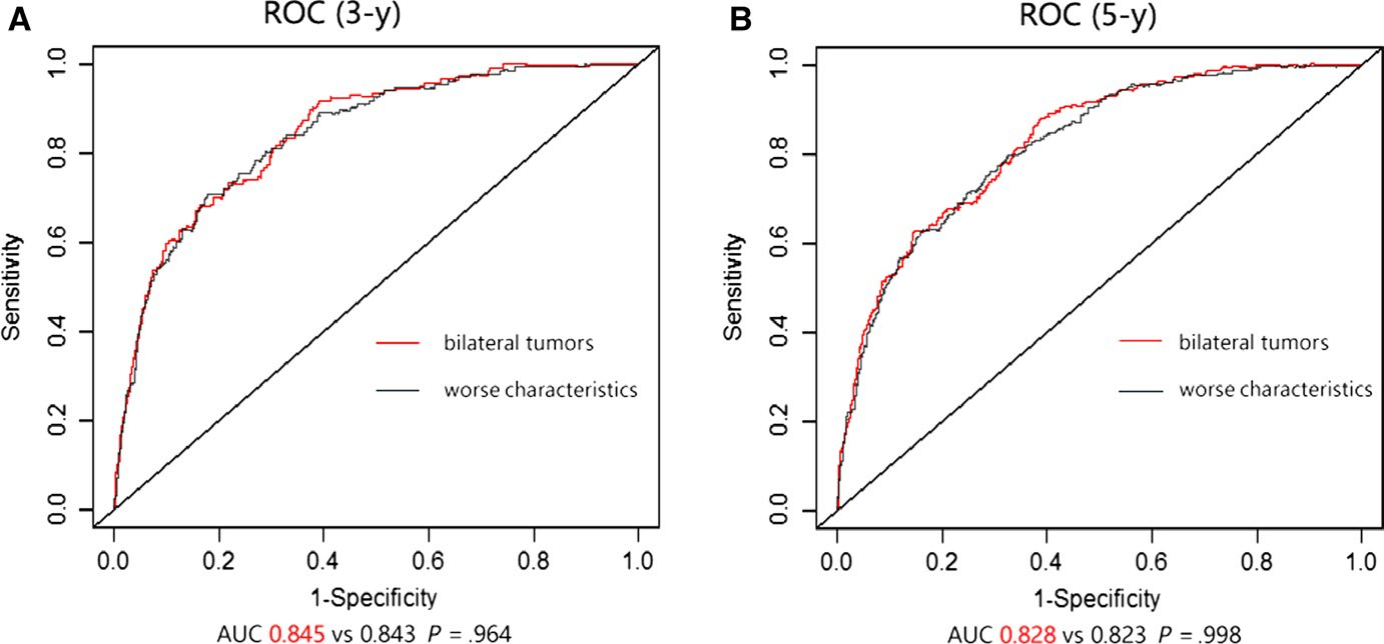
Receiver operating characteristic curves (ROC) for evaluating the performance of predicting 3‐y (A) and 5‐y (B) survival between bilateral tumors and worse characteristics regardless of side in bilateral primary breast cancer

These competing risk nomograms were validated using the validation cohort and internally processed. The calibration plots of two nomograms (the bilateral tumors and the worse characteristics) were presented in Figures 2 and 3, which showed good coordination between the predicted and observed outcomes. Concordance index was calculated in the bilateral tumors (0.819 (95% CI: 0.793‐0.844)) and in the worse characteristics (0.816 (95% CI: 0.791‐0.840)). The two values were almost identical and not statistically different. Concordance index in worse tumor was 0.807 (0.782‐0.832), in first tumor was 0.744 (0.728‐0.763) and in second tumor was 0.778 (0.762‐0.794). In Figure 4, the model of the bilateral tumors was similar to the model of the worse characteristics in 3‐year (AUC: 0.845 vs 0.843; *P* = .964) and 5‐year (AUC: 0.828 vs 0.823; *P* = .998). However, compared with other three models (Figures [Link], [Link], [Link]), the predictive power of bilateral tumors was superior to them (*P* < .05), except for the model of the worse tumor in 5‐year (*P* = .437). Finally, regarding the worse characteristics as a new model, we computed the NRI in 3‐ (2.7%) and 5‐year (−1.0%), which indicated the predictive ability of the new model improved in 3‐year and worsened in 5‐year. Consequently, it also could not explain which model gained advantage.

## 4 DISCUSSION

This study picked out a most convenient and efficient prognostic model out of five to evaluate the mortality for patients with BPBC diagnosed from 2004 to 2014 in the SEER registry. As far as we know, the study is the first to develop a competing risk nomogram and select the worse characteristics as predictive factors, due to the complexity of bilateral variables, to predict survival using a large population‐based cohort based on Fine and Gray's competing risk regression analysis. Moreover, further verification indicated that the model performed well in predicting the survival of 3‐ and 5‐year for BPBC patients.

In virtue of the prolonged lifetime, advanced treatment and early detection through systematic screening, the incidence of BPBC had been rising.^4^, ^24^ Most studies focused on diverse clinical features and outcomes between BPBC and UBC. Also, the majority of authors reported the higher incidence and worse survival of BPBC.^9^, ^25^, ^26^, ^27^, ^28^ However, few studies concentrated on the poor prognostic indicators for survival of BPBC patients. So far, no study has reported about competing risk analysis and nomogram of BPBC.

Because of the high proportion of nonbreast cancer deaths, we have to take advantage of competitive risk analysis to explore prognostic factors accurately and reasonably. The results of our study demonstrated that age, interval time, tumor size, lymph nodes, tumor grade and ER status were closely associated with survival in BPBC patients. It revealed that young age, low tumor grade, small tumor size, ER positivity and no lymph node involvement were significant beneficial prognostic factors for survival of both SBBC and MBBC, no matter in the first tumor or in the second tumor. Several studies strongly supported and gave reassuring evidence.^7^, ^28^, ^29^, ^30^, ^31^ Admittedly, it is recognized that these are also considered as vigorous prognostic factors in UBC patients because BPBC itself is derived from UBC. To some extent, they are the same disease. Why BPBC have inferior prognosis? Most probably, larger overall tumor burden of both sides becomes the reason affects prognosis. Mejdahl et al and Qiu et al found out that the combined effect of having two cancers contributed to excess mortality in BPBC.^29^, ^32^ Our study agreed with the point and regraded bilateral tumors variables as a reference standard in five models. Based on this, we performed comparisons and selected an optimal model.

The definition of SBBC and MBBC in the existing literature is ambiguous. There is no consensus on the definite cutoff time of interval and each author follows a different criterion. According to X‐tile, we chose 4 months as the cutoff time to distinguish SBBC from MBBC. Mejdahl et al shared the similar view on this,^32^ while several studies even used a shorter cutoff time such as 3 months.^33^, ^34^ In comparison with SBBC, MBBC was more often with ER, PR discordance and concordant negativity that resulted in poorer prognosis.^35^ It is possible that ER/PR positive tumor is prone to be treated with endocrine therapy, which greatly reduces the risk of developing BPBC as time goes on.^36^, ^37^ Besides, the first tumor of MBBC had a relatively favorable stage, yet the second tumor of MBBC leaned toward larger tumor size, worse differentiated grade and more axillary nodal involvement. Here is an interpretation for this phenomenon: the invasive neoplasms progress in response to therapy or over time, which lead to heterogeneous diseases.^38^ Several studies held the similar point that MBBC was related to worse survival.^4^, ^26^, ^39^ They also suggested that MBBC was more likely to show local recurrence. Except to heterogeneity of tumors, maybe it was also because that patients with SBBC tended to receive mastectomy instead of breast‐conserving surgery, which was also presented in Table 2 in our study. Patients with MBBC should be followed particularly closely in order to detect recurrence early and maximize quality of life. Moreover, in our study, patients diagnosed with contralateral breast cancer within 1 month showed poorer survival in SBBC (Figure 1; Table 4). These patients perhaps diagnosed within 1 month had a higher tumor burden concurrently that imperiled their survival prospects. Patients may not be able to endure under bilateral tumor load in such a short time.

Undeniably, there are some limitations in our study. Firstly, the SEER database is short of schemes, dosage, frequency and periods of chemotherapy, radiotherapy and endocrine therapy, which might cause result bias. Secondly, due to the limited SEER dataset, family history and HER‐2 status are unable to be included in this study. Thirdly, external validation set was lack to examine interaction competing risk analysis and nomogram. Last but not least, as a retrospective cohort population, inevitable selection bias might affect the conclusions. More large‐scale prospective randomized controlled trials are warranted to identify the risk factors.

In summary, our study found out that age, interval time, bilateral tumor size, bilateral lymph nodes, bilateral tumor grade and bilateral ER status had a strong correlation with survival of BPBC. Thereinto, MBBC (interval > 4 months) presented poorer survival than SBBC (interval ≤ 4 months). In view of these above, a competing risk nomogram were constructed from a new model that incorporated into the worse characteristics regardless of side, which was concise, valid and never mentioned in other literatures. The nomogram may assist clinicians in predicting the survival and evaluating the stage of disease with quantifying indicators in order to guide the management of BPBC patients.

## CONFLICT OF INTEREST

None declared.

## Data Availability

The SEER Program collects data from population‐based cancer registries with anonymous information. The SEER is a publicly available database and data extracted from SEER was deemed “non‐human study” by the North Shore LIJ IRB committee.

## References

[1] Siegel RL , Miller KD , Jemal A . Cancer statistics, 2018. CA Cancer J Clin. 2018;68(1):7‐30. 29313949 10.3322/caac.21442

[2] Soerjomataram I , Louwman WJ , Lemmens VE , de Vries E , Klokman WJ , Coebergh JW . Risks of second primary breast and urogenital cancer following female breast cancer in the south of The Netherlands, 1972–2001. Eur J Cancer. 1990;2005(41):2331‐2337. 10.1016/j.ejca.2005.01.029 16140007

[3] Lu W , Schaapveld M , Jansen L , et al. The value of surveillance mammography of the contralateral breast in patients with a history of breast cancer. Eur J Cancer. 1990;2009(45):3000‐3007. 10.1016/j.ejca.2009.08.007 19744851

[4] Hartman M , Czene K , Reilly M , et al. Incidence and prognosis of synchronous and metachronous bilateral breast cancer. J Clin Oncol. 2007;25:4210‐4216. 17878475 10.1200/JCO.2006.10.5056

[5] Ozturk A , Alco G , Sarsenov D , et al. Synchronous and metachronous bilateral breast cancer: a long‐term experience. J BUON. 2018;23:1591‐1600. 30610782

[6] Font‐Gonzalez A , Liu L , Voogd AC , et al. Inferior survival for young patients with contralateral compared to unilateral breast cancer: a nationwide population‐based study in the Netherlands. Breast Cancer Res Treat. 2013;139:811‐819. 23760860 10.1007/s10549-013-2588-9

[7] Vichapat V , Garmo H , Holmberg L , et al. Prognosis of metachronous contralateral breast cancer: importance of stage, age and interval time between the two diagnoses. Breast Cancer Res Treat. 2011;130:609‐618. 21671018 10.1007/s10549-011-1618-8

[8] Vichapat V , Garmo H , Holmqvist M , et al. Tumor stage affects risk and prognosis of contralateral breast cancer: results from a large Swedish‐population‐based study. J Clin Oncol. 2012;30:3478‐3485. 22927521 10.1200/JCO.2011.39.3645

[9] Langballe R , Frederiksen K , Jensen M‐B , et al. Mortality after contralateral breast cancer in Denmark. Breast Cancer Res Treat. 2018;171:489‐499. 29948403 10.1007/s10549-018-4846-3

[10] Berry DA , Cronin KA , Plevritis SK , et al. Effect of screening and adjuvant therapy on mortality from breast cancer. N Engl J Med. 2005;353:1784‐1792. 16251534 10.1056/NEJMoa050518

[11] Miller KD , Siegel RL , Lin CC , et al. Cancer treatment and survivorship statistics, 2016. CA Cancer J Clin. 2016;66:271‐289. 27253694 10.3322/caac.21349

[12] Zagar TM , Cardinale DM , Marks LB . Breast cancer therapy‐associated cardiovascular disease. Nat Rev Clin Oncol. 2016;13:172‐184. 26598943 10.1038/nrclinonc.2015.171

[13] Simpson MC , Massa ST , Boakye EA , et al. Primary cancer vs competing causes of death in survivors of head and neck cancer. JAMA Oncol. 2018;4(2):257. 29285537 10.1001/jamaoncol.2017.4478 PMC5838703

[14] Eguchi T , Bains S , Lee M‐C , et al. Impact of increasing age on cause‐specific mortality and morbidity in patients with stage I non‐small‐cell lung cancer: a competing risks analysis. J Clin Oncol. 2017;35:281‐290. 28095268 10.1200/JCO.2016.69.0834 PMC5456376

[15] Zhang J , Peng H , Chen L , et al. Decreased overall and cancer‐specific mortality with neoadjuvant chemotherapy in locoregionally advanced nasopharyngeal carcinoma treated by intensity‐modulated radiotherapy: multivariate competing risk analysis. J Cancer. 2017;8:2587‐2594. 28900496 10.7150/jca.20081 PMC5595088

[16] Kutikov A , Egleston BL , Wong Y‐N , Uzzo RG . Evaluating overall survival and competing risks of death in patients with localized renal cell carcinoma using a comprehensive nomogram. J Clin Oncol. 2010;28:311‐317. 19933918 10.1200/JCO.2009.22.4816 PMC2815719

[17] Yang L , Shen W , Sakamoto N . Population‐based study evaluating and predicting the probability of death resulting from thyroid cancer and other causes among patients with thyroid cancer. J Clin Oncol. 2013;31(4):468‐474. 23270002 10.1200/JCO.2012.42.4457

[18] Zhou H , Zhang Y , Qiu Z , et al. Nomogram to predict cause‐specific mortality in patients with surgically resected stage I non‐small‐cell lung cancer: a competing risk analysis. Clin Lung Cancer. 2018;19:e195‐e203. 29153966 10.1016/j.cllc.2017.10.016

[19] Balachandran VP , Gonen M , Smith JJ , DeMatteo RP . Nomograms in oncology: more than meets the eye. Lancet Oncol. 2015;16:e173‐e180. 25846097 10.1016/S1470-2045(14)71116-7 PMC4465353

[20] Carmona R , Zakeri K , Green G , et al. Improved method to stratify elderly patients with cancer at risk for competing events. J Clin Oncol. 2016;34:1270‐1277. 26884579 10.1200/JCO.2015.65.0739 PMC5070568

[21] Fine JP , Gray RJ . A proportional hazards model for the subdistribution of a competing risk. J Am Stat Assoc. 1999;94:496‐509.

[22] Harrell FE , Lee KL , Mark DB . Multivariable prognostic models: issues in developing models, evaluating assumptions and adequacy, and measuring and reducing errors. Stat Med. 1996;15:361‐387. 8668867 10.1002/(SICI)1097-0258(19960229)15:4<361::AID-SIM168>3.0.CO;2-4

[23] Pencina MJ , D'Agostino RB , Steyerberg EW . Extensions of net reclassification improvement calculations to measure usefulness of new biomarkers. Stat Med. 2011;30:11‐21. 21204120 10.1002/sim.4085 PMC3341973

[24] Holm M , Tjonneland A , Balslev E , Kroman N . Prognosis of synchronous bilateral breast cancer: a review and meta‐analysis of observational studies. Breast Cancer Res Treat. 2014;146:461‐475. 25007962 10.1007/s10549-014-3045-0

[25] Quan G , Pommier SJ , Pommier RF . Incidence and outcomes of contralateral breast cancers. Am J Surg. 2008;195(5):645‐650; discussion 50. 18424281 10.1016/j.amjsurg.2008.01.007

[26] Beckmann KR , Buckingham J , Craft P , et al. Clinical characteristics and outcomes of bilateral breast cancer in an Australian cohort. Breast. 2011;20:158‐164. 21093260 10.1016/j.breast.2010.10.004

[27] Jobsen JJ , van der Palen J , Ong F , Riemersma S , Struikmans H . Bilateral breast cancer, synchronous and metachronous; differences and outcome. Breast Cancer Res Treat. 2015;153:277‐283. 26268697 10.1007/s10549-015-3538-5

[28] Kheirelseid EAH , Jumustafa H , Miller N , et al. Bilateral breast cancer: analysis of incidence, outcome, survival and disease characteristics. Breast Cancer Res Treat. 2011;126:131‐140. 20665107 10.1007/s10549-010-1057-y

[29] Qiu R , Zhao W , Yang J , et al. Comparative analysis of outcomes and clinicopathological characteristics of synchronous and metachronous contralateral breast cancer: a study of the SEER database. J Breast Cancer. 2019;22(2):297‐310. 31281731 10.4048/jbc.2019.22.e18 PMC6597405

[30] Ibrahim NY , Sroor MY , Darwish DO . Impact of bilateral breast cancer on prognosis: synchronous versus metachronous tumors. Asian Pac J Cancer Prev. 2015;16:1007‐1010. 25735321 10.7314/apjcp.2015.16.3.1007

[31] Marmor S , Portschy PR , Burke EE , Virnig BA , Tuttle TM . Prognostic factors for metachronous contralateral breast cancer: implications for management of the contralateral breast. Breast J. 2017;23:299‐306. 27988977 10.1111/tbj.12732

[32] Mejdahl MK , Wohlfahrt J , Holm M , et al. Breast cancer mortality in synchronous bilateral breast cancer patients. Br J Cancer. 2019;120:761‐767. 30804429 10.1038/s41416-019-0403-z PMC6461871

[33] Schmid SM , Pfefferkorn C , Myrick ME , et al. Prognosis of early‐stage synchronous bilateral invasive breast cancer. Eur J Surg Oncol. 2011;37:623‐628. 21628090 10.1016/j.ejso.2011.05.006

[34] Díaz R , Munárriz B , Santaballa A , Palomar L , Montalar J . Synchronous and metachronous bilateral breast cancer: a long‐term single‐institution experience. Med Oncol. 2012;29(1):16‐24. 21193967 10.1007/s12032-010-9785-8

[35] Baretta Z , Olopade OI , Huo D . Heterogeneity in hormone‐receptor status and survival outcomes among women with synchronous and metachronous bilateral breast cancers. Breast. 2015;24:131‐136. 25534718 10.1016/j.breast.2014.12.001 PMC4375038

[36] Mellemkjær L , Steding‐Jessen M , Frederiksen K , et al. Risk of contralateral breast cancer after tamoxifen use among Danish women. Ann Epidemiol. 2014;24:843‐848. 25277504 10.1016/j.annepidem.2014.08.003

[37] Aihara T , Tanaka S , Sagara Y , et al. Incidence of contralateral breast cancer in Japanese patients with unilateral minimum‐risk primary breast cancer, and the benefits of endocrine therapy and radiotherapy. Breast Cancer. 2014;21(3):284‐291. 23054843 10.1007/s12282-012-0396-4

[38] Balko JM , Giltnane JM , Wang K , et al. Molecular profiling of the residual disease of triple‐negative breast cancers after neoadjuvant chemotherapy identifies actionable therapeutic targets. Cancer Discov. 2014;4:232‐245. 24356096 10.1158/2159-8290.CD-13-0286 PMC3946308

[39] Kuo W‐H , Yen A M‐F , Lee P‐H , et al. Taiwanese women. Br J Cancer. 1907;2009(100):563‐570. 10.1038/sj.bjc.6604898 PMC2653740 19190627

