# Supplementary figures and images for: Worse characteristics can predict survival effectively in bilateral primary breast cancer: A competing risk nomogram using the SEER database
Source: Cancer Med. 2019 Oct 30;8(18):7890–902. doi: 10.1002/cam4.2662 (PMC6912037; doi:10.1002/cam4.2662)

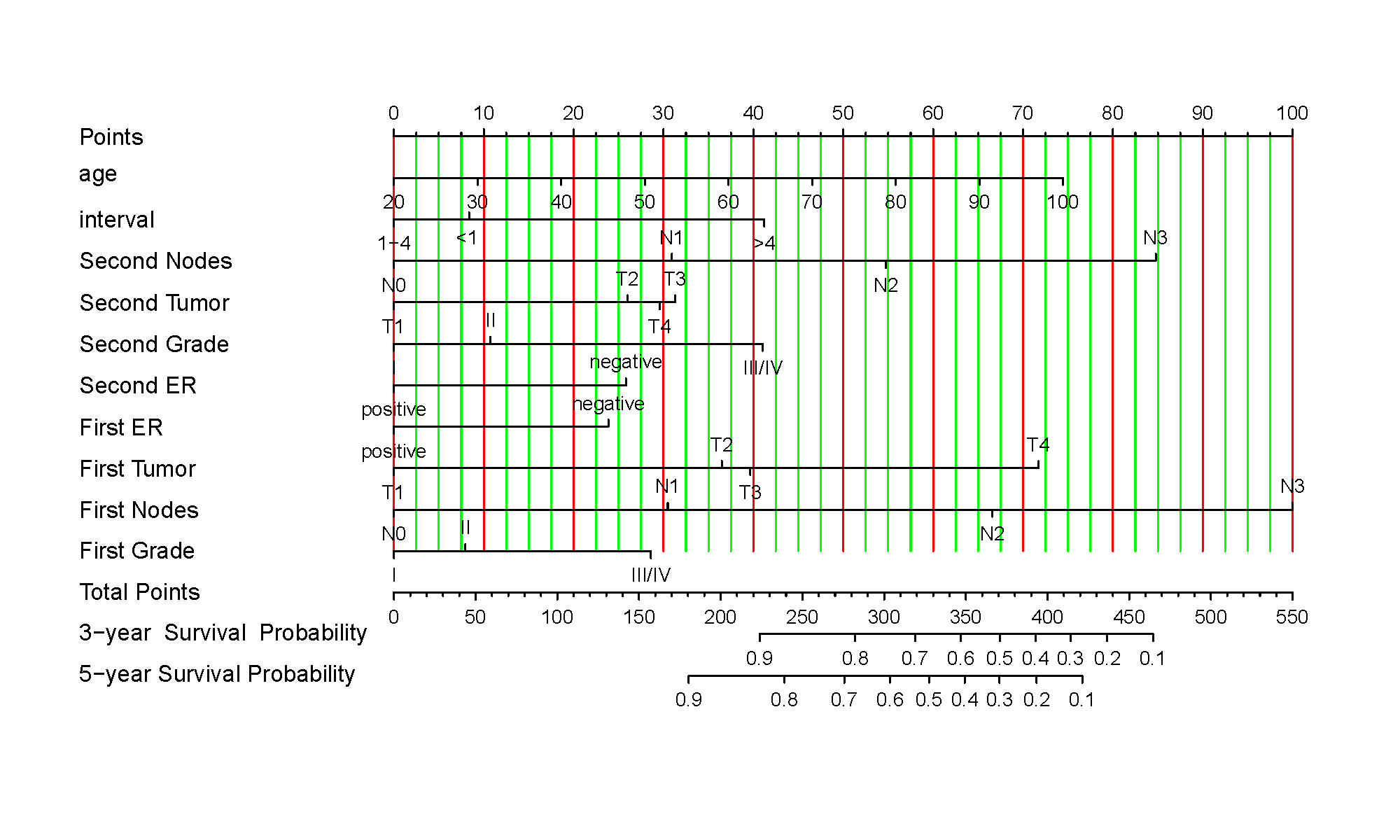

Supplement: Supplementary file 1 [file CAM4-8-7890-s001.tiff]

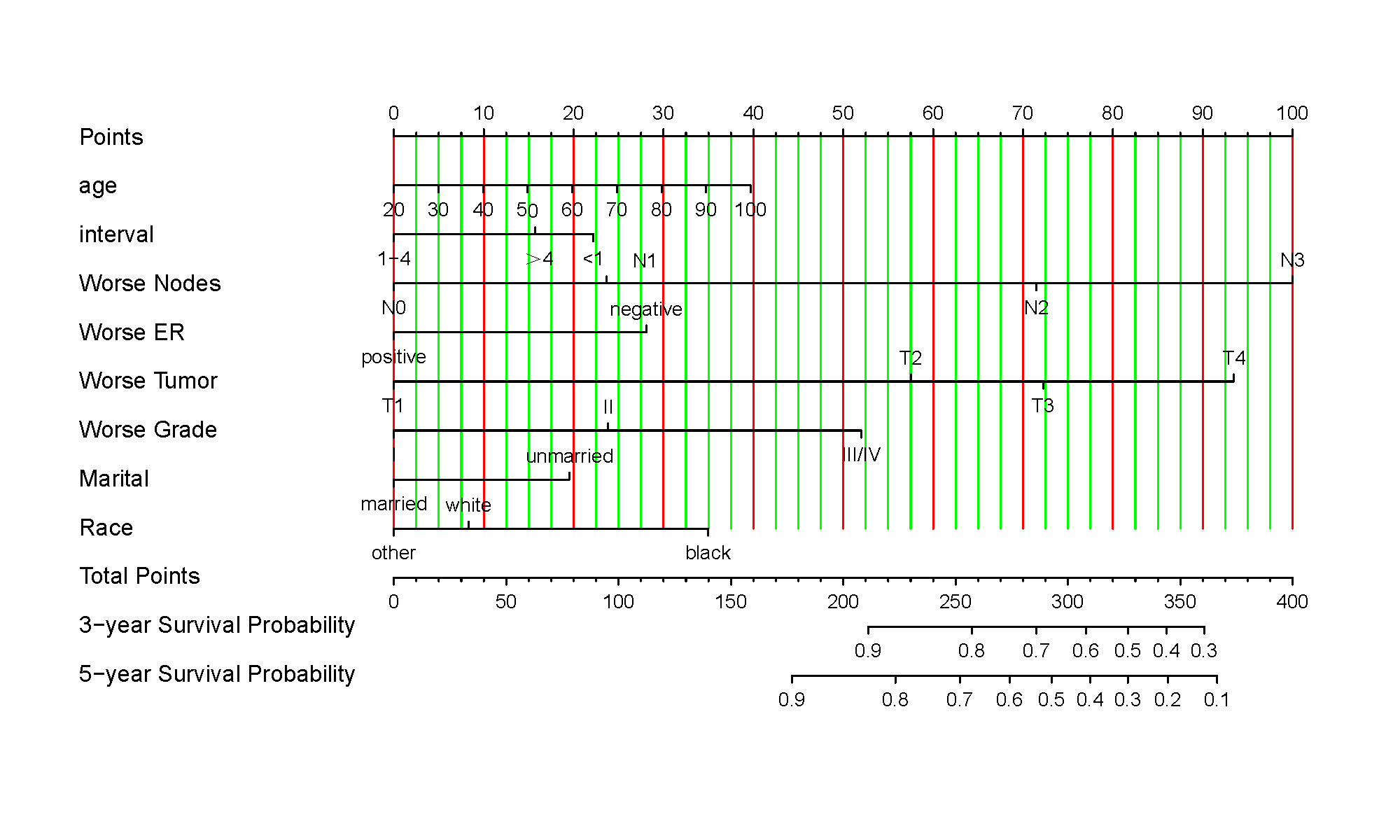

Supplement: Supplementary file 2 [file CAM4-8-7890-s002.tif]

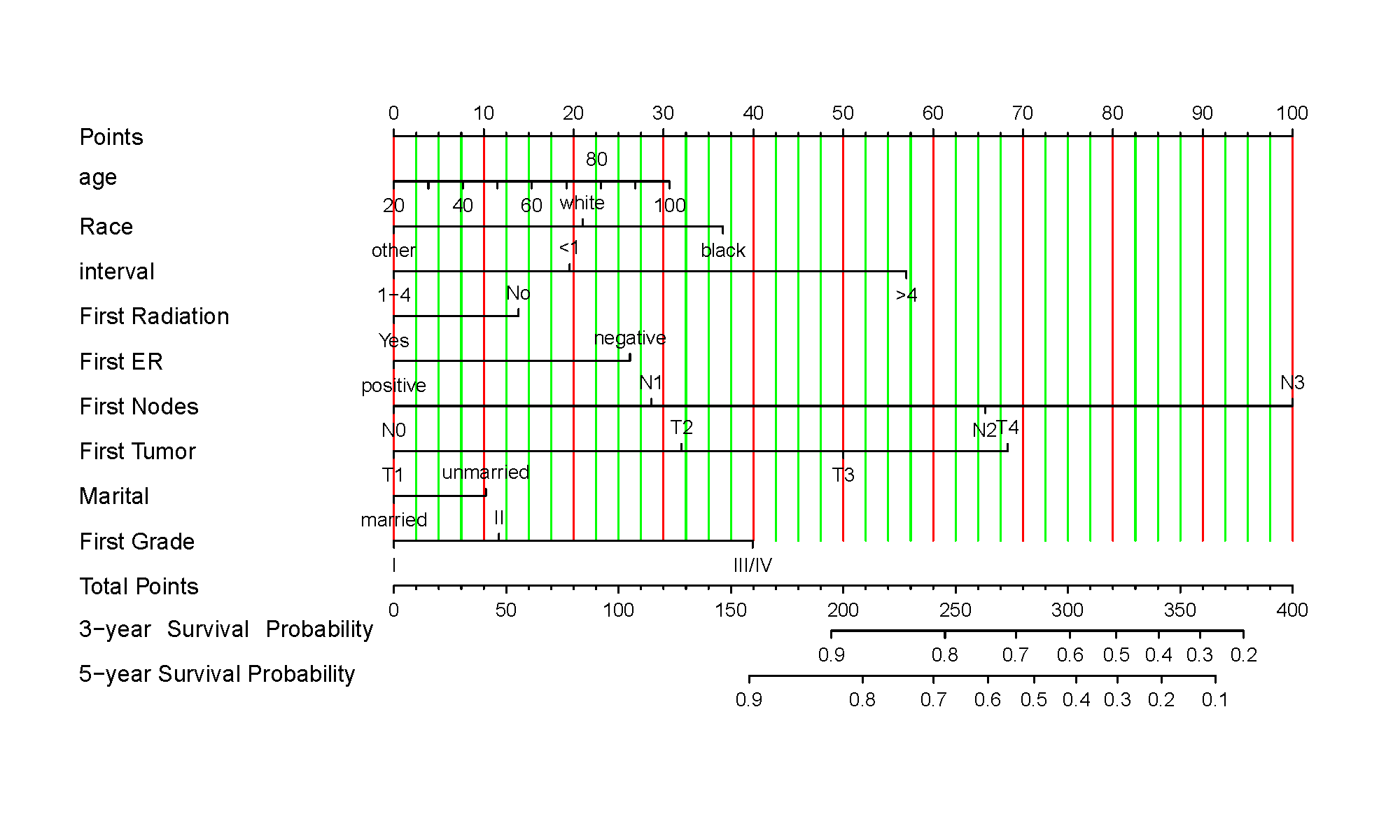

Supplement: Supplementary file 3 [file CAM4-8-7890-s003.tiff]

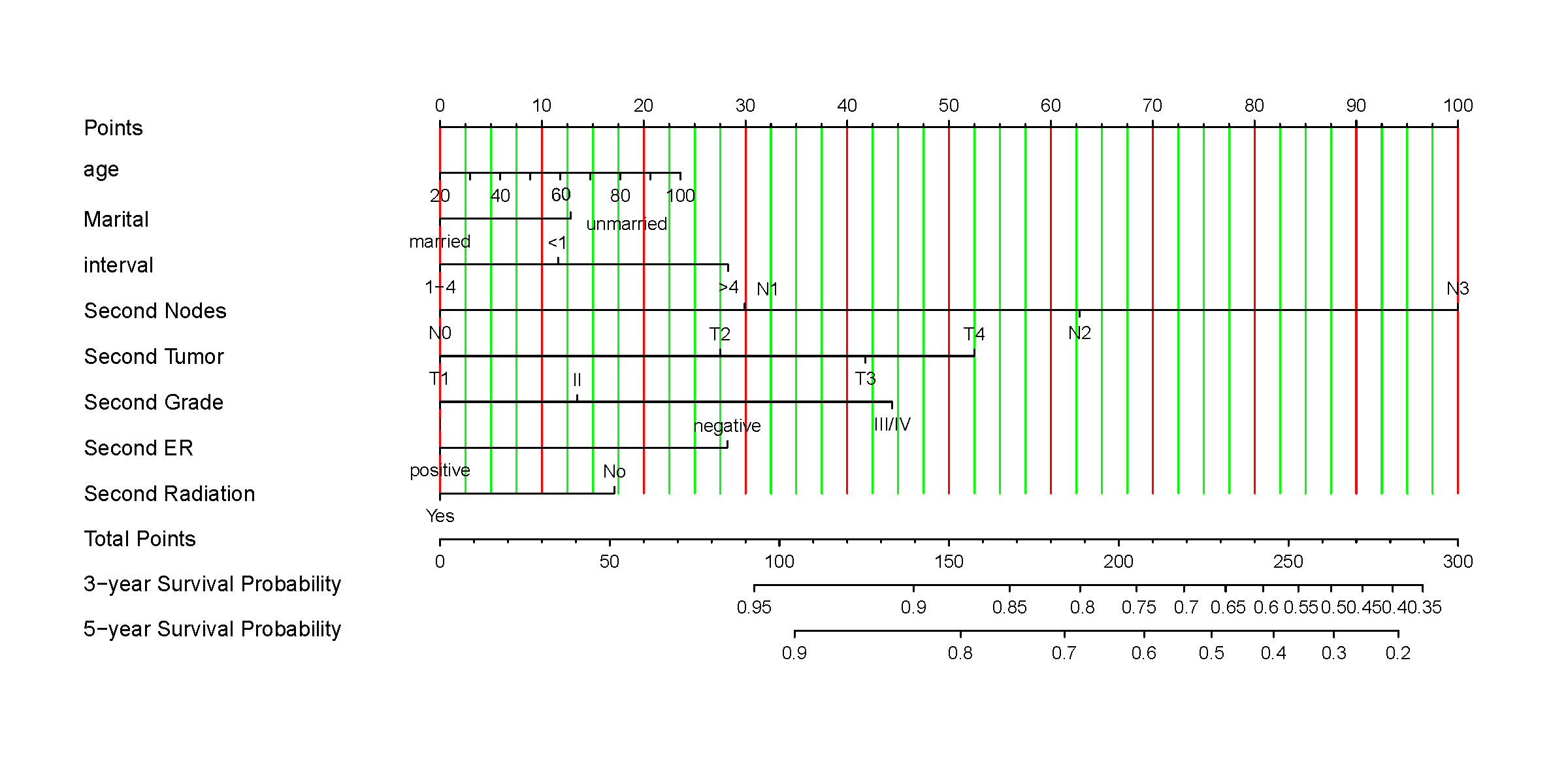

Supplement: Supplementary file 4 [file CAM4-8-7890-s004.tiff]

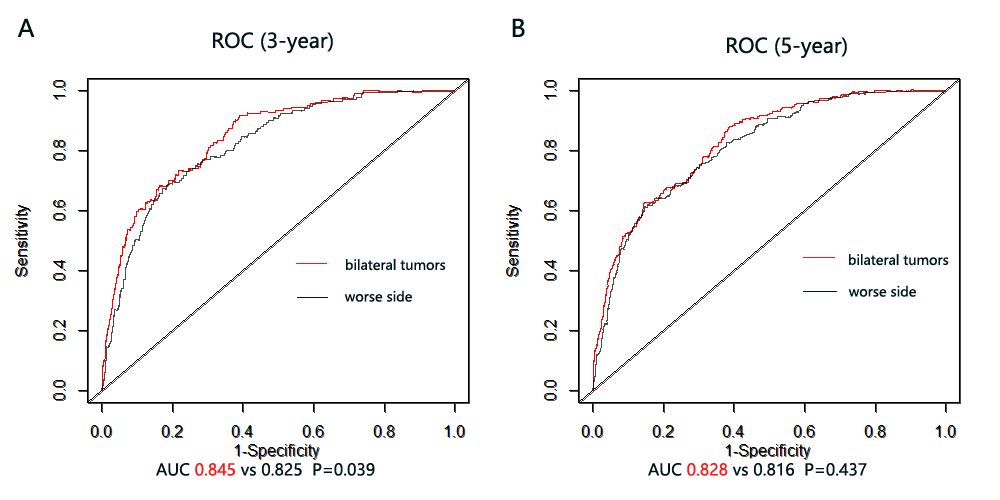

Supplement: Supplementary file 5 [file CAM4-8-7890-s005.tif]

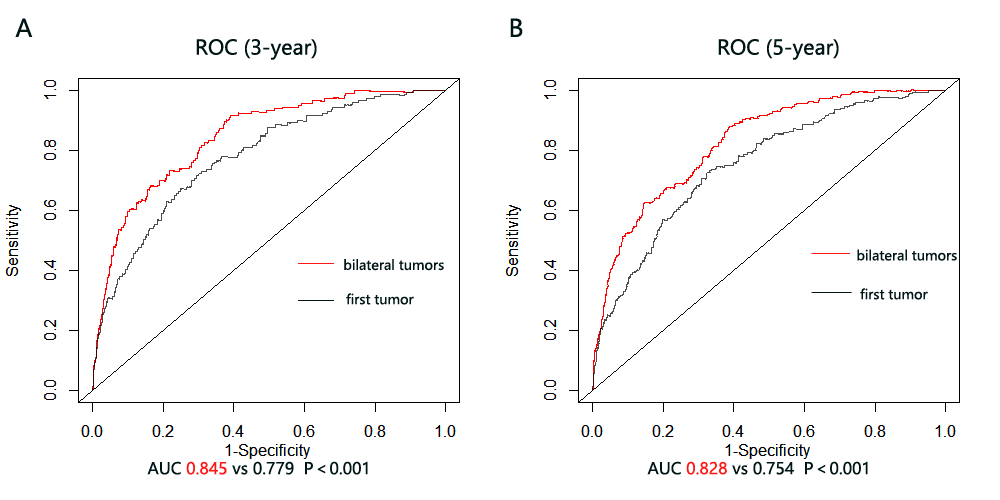

Supplement: Supplementary file 6 [file CAM4-8-7890-s006.tif]

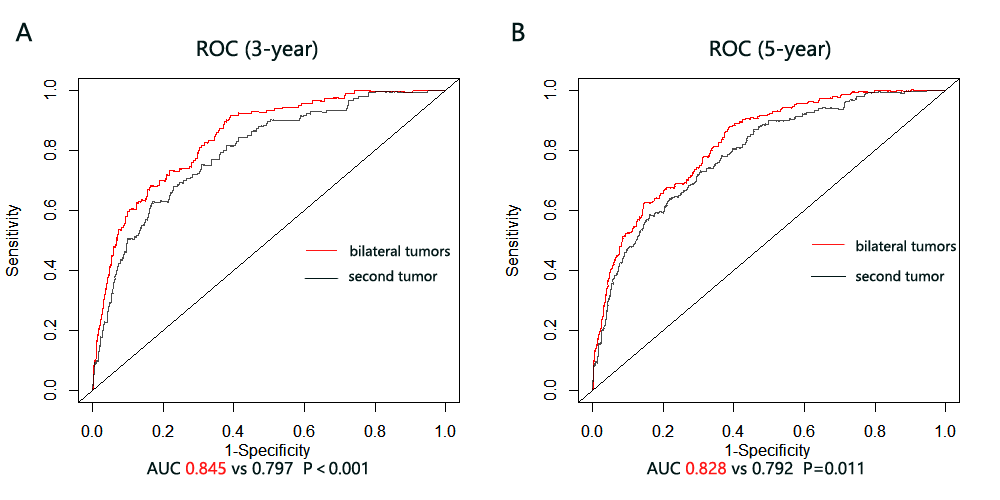

Supplement: Supplementary file 7 [file CAM4-8-7890-s007.tif]
