# Supplementary material for: Worse characteristics can predict survival effectively in bilateral primary breast cancer: A competing risk nomogram using the SEER database
Source: Cancer Med. 2019 Oct 30;8(18):7890–902. doi: 10.1002/cam4.2662 (PMC6912037; doi:10.1002/cam4.2662)
Supplement: Supplementary file 8 [file CAM4-8-7890-s008.docx]

Table S1: Multivariate COX and competing risk analysis of characteristics of worse side in BPBC.

| variable | Multivariate Analysis | | Multivariable Competing Risk Analysis | |
| --- | --- | --- | --- | --- |
|  | HR(95%CI) | P-value | SHR(95%CI) | P-value |
| Age | 1.013(1.003-1.023) | 0.001 | 1.008(0.997-1.019) | 0.015 |
| Race |  |  |  |  |
| White | reference |  | reference |  |
| Black | 1.5626(1.118-2.185) | 0.0091 | 1.556(1.102-2.195) | 0.012 |
| Other | 0.841(0.494-1.432) | 0.5239 | 0.877(0.520-1.478) | 0.620 |
| Marital |  |  |  |  |
| Yes | reference |  | reference |  |
| No | 1.398(1.073-1.821) | <0.001 | 1.361（1.045-1.773） | 0.022 |
| Interval (months) |  |  |  |  |
| <1 | reference |  | reference |  |
| 1-4 | 0.684(0.483-0.969) | 0.033 | 0.696(0.460-0.989) | 0.043 |
| >4 | 0.925(0.69-1.241) | 0.603 | 0.917(0.675-1.247) | 0.58 |
| Worse Tumor size |  |  |  |  |
| T1 | reference |  | reference |  |
| T2 | 2.588(1.181-3.698) | <0.001 | 2.485(1.728-3.575) | <0.001 |
| T3 | 3.344(2.105-5.312) | <0.001 | 3.186(1.992-5.095) | <0.001 |
| T4 | 4.997(3.249-7.686) | <0.001 | 4.469(2.814-7.096) | <0.001 |
| Worse Lymph Nodes |  |  |  |  |
| N0 | reference |  | reference |  |
| N1 | 1.464(1.0522-2.037) | <0.001 | 1.452(1.036-2.036) | 0.03 |
| N2 | 3.275(2.259-4.747) | <0.001 | 3.195(2.166-4.713) | <0.001 |
| N3 | 5.357(3.630-7.907) | <0.001 | 5.025(3.348-7.543) | <0.001 |
| Grade |  |  |  |  |
| I | reference |  | reference |  |
| II | 1.492(0.906-2.458) | 0.116 | 1.486(0.913-2.419) | 0.110 |
| III/IV | 2.371(1.429-3.933) | <0.001 | 2.421(1.482-3.955) | <0.001 |
| Worse ER |  |  |  |  |
| Positive | reference |  | reference |  |
| Negative | 1.589(1.195-2.113) | 0.0014 | 1.361(1.045-1.773) | 0.004 |

After step wise model selection, we excluded bilateral histologic, bilateral surgery, PR and radiation. BPBC, bilateral primary breast cancer; ER, estrogen receptor; PR, progesterone receptor.
