# Supplementary material for: Worse characteristics can predict survival effectively in bilateral primary breast cancer: A competing risk nomogram using the SEER database
Source: Cancer Med. 2019 Oct 30;8(18):7890–902. doi: 10.1002/cam4.2662 (PMC6912037; doi:10.1002/cam4.2662)
Supplement: Supplementary file 9 [file CAM4-8-7890-s009.docx]

Table S2: Multivariate COX and competing risk analysis of first tumor in BPBC.

| variable | Multivariate Analysis | | Multivariable Competing Risk Analysis | |
| --- | --- | --- | --- | --- |
|  | HR(95%CI) | P-value | SHR(95%CI) | P-value |
| Age | 1.011(1.005-1.017) | <0.001 | 1.006(1.000-1.012) | 0.052 |
| Race |  |  |  |  |
| White | reference |  | reference |  |
| Black | 1.342(1.086-1.657) | 0.006 | 1.261(1.005-1.584) | 0.046 |
| Other | 0.727(0.531-0.996) | 0.047 | 0.732(0.532-1.007) | 0.056 |
| Marital |  |  |  |  |
| Yes | reference |  | reference |  |
| No | 1.227(1.056-1.425) | 0.007 | 1.164（1.001-1.353） | 0.048 |
| Interval (months) |  |  |  |  |
| <1 | reference |  | reference |  |
| 1-4 | 0.748(0.603-0.928) | 0.008 | 0.748(0.604-0.926) | 0.008 |
| >4 | 1.846(1.5607-2.1832) | <0.001 | 1.743(1.464-2.074) | <0.001 |
| First Tumor size |  |  |  |  |
| T1 | reference |  | reference |  |
| T2 | 1.633（1.361-1.958) | <0.001 | 1.608(1.343-1.927) | <0.001 |
| T3 | 2.168(1.643-2.860) | <0.001 | 2.100(1.581-2.788) | <0.001 |
| T4 | 3.050(2.392-3.890) | <0.001 | 2.753(2.134-3.551) | <0.001 |
| First Lymph Nodes |  |  |  |  |
| N0 | reference |  | reference |  |
| N1 | 1.548(1.279-1.873) | <0.001 | 1.530(1.262-1.856) | <0.001 |
| N2 | 2.669(2.127-3.348) | <0.001 | 2.650(2.092-3.357) | <0.001 |
| N3 | 4.414(3.445-5.655） | <0.001 | 4.406(3.386-5.732) | <0.001 |
| First Grade |  |  |  |  |
| I | reference |  | reference |  |
| II | 1.209(0.961-1.521) | 0.105 | 1.189(0.948-1.493) | 0.130 |
| III/IV | 1.897(1.492-2.412) | <0.001 | 1.809(1.422-2.301) | <0.001 |
| First Radiation |  |  |  |  |
| Yes | reference |  |  |  |
| No | 1.283(1.105-1.490) | 0.001 | 1.228(1.054-1.430) | 0.008 |
| First ER |  |  |  |  |
| Positive | reference |  | reference |  |
| Negative | 1.468(1.246-1.729) | <0.001 | 1.476(1.244-1.752) | <0.001 |

After wise model selection: we excluded: first histologic, first PR and first surgery. BPBC, bilateral primary breast cancer; BCS, breast-conserving surgery; ER, estrogen receptor; PR, progesterone receptor.
