# Supplementary material for: Worse characteristics can predict survival effectively in bilateral primary breast cancer: A competing risk nomogram using the SEER database
Source: Cancer Med. 2019 Oct 30;8(18):7890–902. doi: 10.1002/cam4.2662 (PMC6912037; doi:10.1002/cam4.2662)
Supplement: Supplementary file 10 [file CAM4-8-7890-s010.docx]

Table S3: Multivariate COX and competing risk analysis of second tumor in BPBC.

| variable | Multivariate Analysis | | Multivariable Competing Risk Analysis | |
| --- | --- | --- | --- | --- |
|  | HR(95%CI) | P-value | SHR(95%CI) | P-value |
| Age | 1.010(1.004-1.016) | <0.001 | 1.005(0.999-1.01) | 0.001 |
| Race |  |  |  |  |
| White | reference |  | reference |  |
| Black | 1.278(1.034-1.579) | 0.023 | 1.202(0.956-1.51) | 0.120 |
| Other | 0.7129(0.521-0.976) | 0.034 | 0.727(0.529-1.00) | 0.050 |
| Marital |  |  |  |  |
| Yes | reference |  | reference |  |
| No | 1.252(1.078-1.454) | 0.003 | 1.179（1.015-1.37） | 0.032 |
| Interval (months) |  |  |  |  |
| <1 | reference |  | reference |  |
| 1-4 | 0.829(0.667-1.030) | 0.091 | 0.832(0.667-1.04) | 0.100 |
| >4 | 1.3472(1.138-1.594) | <0.001 | 1.280(1.074-1.53) | <0.001 |
| Second Tumor size |  |  |  |  |
| T1 | reference |  | reference |  |
| T2 | 1.577（1.325-1.877) | <0.001 | 1.521(1.274-1.82) | <0.001 |
| T3 | 1.933(1.419-2.633) | <0.001 | 1.874(1.354-2.59) | <0.001 |
| T4 | 2.504(1.712-3.661) | <0.001 | 2.202(1.469-3.30) | <0.001 |
| Second Lymph Nodes |  |  |  |  |
| N0 | reference |  | reference |  |
| N1 | 1.563(1.290-1.894) | <0.001 | 1.577(1.294-1.92) | <0.001 |
| N2 | 2.658(2.031-3.479) | <0.001 | 2.619(1.962-3.50) | <0.001 |
| N3 | 5.157(3.891-6.833） | <0.001 | 4.559(3.374-6.16) | <0.001 |
| Second Grade |  |  |  |  |
| I | reference |  | reference |  |
| II | 1.230(1.002-1.509) | 0.048 | 1.229(1.003-1.50) | 0.047 |
| III/IV | 1.964(1.573-2.453) | <0.001 | 1.943(1.553-2.43) | <0.001 |
| Second Radiation |  |  |  |  |
| Yes | reference |  |  |  |
| No | 1.377(1.172-1.618) | <0.001 | 1.308(1.108-1.54) | 0.002 |
| Second ER |  |  |  |  |
| Positive | reference |  | reference |  |
| Negative | 1.532(1.300-1.806) | <0.001 | 1.525(1.282-1.81) | <0.001 |

After wise model selection: we excluded second histologic, second PR and second surgery. BPBC, bilateral primary breast cancer; BCS, breast-conserving surgery; ER, estrogen receptor; PR, progesterone receptor.
