# Supplementary material for: Worse characteristics can predict survival effectively in bilateral primary breast cancer: A competing risk nomogram using the SEER database
Source: Cancer Med. 2019 Oct 30;8(18):7890–902. doi: 10.1002/cam4.2662 (PMC6912037; doi:10.1002/cam4.2662)
Supplement: Supplementary file 11 [file CAM4-8-7890-s011.docx]

FIGURE S1: Nomogram for predicting the 3- and 5-year survival probabilities of bilateral tumors in bilateral primary breast cancer. A vertical straight line was drawn from the variable value to the axis labeled “Points” to identify points for each variable. All points were summed, and the total points was projected to the scales along the bottom of the figure that correspond to the 3- and 5-year survival. Notes: ER, estrogen receptor.

FIGURE S2: Nomogram for predicting the 3- and 5-year survival probabilities of characteristics of worse tumor in bilateral primary breast cancer. A vertical straight line was drawn from the variable value to the axis labeled “Points” to identify points for each variable. All points were summed, and the total points was projected to the scales along the bottom of the figure that correspond to the 3- and 5-year survival. Notes: ER, estrogen receptor.

FIGURE S3: Nomogram for predicting the 3- and 5-year survival probabilities of first tumor in bilateral primary breast cancer. A vertical straight line was drawn from the variable value to the axis labeled “Points” to identify points for each variable. All points were summed, and the total points was projected to the scales along the bottom of the figure that correspond to the 3- and 5-year survival. Notes: ER, estrogen receptor.

FIGURE S4: Nomogram for predicting the 3- and 5-year survival probabilities of second tumor in bilateral primary breast cancer. A vertical straight line was drawn from the variable value to the axis labeled “Points” to identify points for each variable. All points were summed, and the total points was projected to the scales along the bottom of the figure that correspond to the 3- and 5-year survival. Notes: ER, estrogen receptor.

FIGURE S5: Receiver operating characteristic curves (ROC) for evaluating the performance of predicting 3-year (A) and 5-year (B) survival between bilateral tumors and the characteristics of worse tumor.

FIGURE S6: Receiver operating characteristic curves (ROC) for evaluating the performance of predicting 3-year (A) and 5-year (B) survival between bilateral tumors and first tumor.

FIGURE S7: Receiver operating characteristic curves (ROC) for evaluating the performance of predicting 3-year (A) and 5-year (B) survival between bilateral tumors and second tumor.
